# Editorial: Public health promotion in university students

**DOI:** 10.3389/fpubh.2022.993982

**Published:** 2022-08-22

**Authors:** Pavel Dietz, Estelle D. Watson, Mireille van Poppel, Ana N. Tibubos

**Affiliations:** ^1^Institute of Occupational, Social and Environmental Medicine, University Medical Centre of the University of Mainz, Mainz, Germany; ^2^Department of Exercise Science, Faculty of Science, University of Auckland, Auckland, New Zealand; ^3^Institute of Human Movement Science, Sport and Health, University of Graz, Graz, Austria; ^4^Diagnostics in Healthcare and E-Health, University of Trier, Trier, Germany

**Keywords:** college, campus, tertiary education, health education, setting approach

## Introduction

According to the World Health Organization, health is a state of complete physical, mental, and social wellbeing and not merely the absence of disease or infirmity. It is further described as “a resource for everyday life” which is created and experienced by people within the settings of their everyday life: where they learn, work, play, and love (1). This emphasizes the interconnectedness between individuals and their environments. One important setting of everyday life for health promotion is universities. Here, the collective of university students is particularly relevant, since they are the leaders, decision-makers, and parents of tomorrow (2). Therefore, promoting the health of university students could be sustainable and beneficial for the general society. In addition, a students' entrance into this new living environment frequently causes significant changes in the home, work, and recreational environment (3). Furthermore, according to numerous studies, the time of young adulthood (18–25 years) has been characterized as a critical period where people are potentially vulnerable for engaging in risky health behaviors such as drinking, drug use or physical inactivity (4).

Although a large number of studies has been performed in this field, there are still under investigated topics which need more attention. For example, according to a systematic umbrella review by our group, including 81 systematic reviews and meta-analyses, mental health, and alcohol consumption are well-investigated among university students, whereas studies on topics such as sleep hygiene or media consumption are rare (5). Furthermore, the COVID-19 pandemic led to drastic changes in university student's life and conditions of studying. For example, closing of universities led to an abrupt loss of personal contacts with peers and faculty, postponement of curricula, research, practical work, and exchange programs (6, 7). In addition, the abrupt and often ill-prepared switch to online learning may have led to stress among students (8). Finally, the loss of temporary jobs due to pandemic-related reasons could have compounded financial uncertainties (9).

Therefore, the aim of the current Research Topic is to address these gaps and to provide a Research Topic of up-to-date and high quality research papers focusing on the effects of health-promoting interventions as well as the epidemiology of health (not limited to health behavior only) in university students with focus on, but not limited to, the topics of media consumption, sleep hygiene, nutrition, physical inactivity, sedentary behavior, mental health, and the effect of the COVID-19 pandemic on university students life and health. In order to develop and implement evidence-based health-promoting interventions, it is further necessary to investigate potential correlates (factors that are associated with) or determinants (factors with a causal relationship) of health and health behavior.

## Content of the Research Topic

Overall, 22 papers were submitted to the Research Topic of which 14 were accepted for publication after review process (rejection rate: 36%). Four papers referred to health aspects during the COVID-19 pandemic. Schäfer et al. investigated health information seeking among university students before and during the pandemic taking cross-sectional as well as longitudinal data form two online surveys conducted in Germany into account. Furthermore, Defeyter et al. and Matos Fialho et al. focused in their empirical studies conducted in UK higher education students and university students in Germany on mental wellbeing during the pandemic. Both came to the conclusion that a significant proportion of university students faced low levels of mental wellbeing during the pandemic, underlining the need for universities to provide intervention strategies targeting students' mental wellbeing during the pandemic. Finally, Dietz et al. compared the prevalence of pharmacological neuroenhancement (PN) among university students in Germany before and during the pandemic analyzing three consecutive cross-sectional survey studies (one before, two during the pandemic). Although the prevalence slightly decreased during the pandemic, they concluded that the fairly high prevalence of PN of around 8% in 2021 demonstrates a persistent urgent need for prevention initiatives to combat the use of PN among university students.

The remaining 10 papers had no specific COVID-19 focus. Within their conceptual paper, Reichel et al. provided an example on how to conduct a health survey at a large campus university in Germany highlighting potential pitfalls and presenting practical recommendations for future empirical studies. Four studies investigated aspects of specific health-related behavior, three with focus on drug use. Franke et al. showed in an online survey among German students that nearly all students use over-the-counter substances such as coffee, caffeinated drinks, energy drinks, and caffeine pills for enhancing their cognitive performance, whereas the use of illegal and prescription substances for this purpose was only 1.8%. By performing a cluster-controlled trial conducted at eight universities in Germany, Pischke et al. showed beneficial effects of a web-based social norms-intervention on alcohol and cannabis use but no intervention effects on tobacco use and episodes of drunkenness. Comparable results were presented by Wolter et al. who concluded that personalized, gender-specific, and selective normative feedback is effective for alcohol prevention among university students. Furthermore, Edelmann et al. assessed physical activity and sedentary behavior in a sample of university students in Germany and performed subgroup-analyses with regard to gender, age, field of study, targeted degree, and study semester to identify student populations with a potential higher risk for decreased physical activity and increased sedentary behavior levels.

Using longitudinal data from three surveys conducted in university students in Germany, Gusy et al. showed that time pressure predicted burnout which, in turn, predicted student's health-related loss of productivity. The paper from Limarutti et al. put specifically 1st year students from an Austrian University of Applied Sciences into focus by evaluating a tailored multi-component onboarding intervention program. They underline the relevance of starting initiatives to promote students' health early at the beginning of studies and the role of students as future multipliers for health promotion and prevention. Two papers had a closer look at structural conditions of the institution university. Using network analysis, Bachert et al. provided in-depth insights into university structures promoting students' health comprising 33 organizational units. They concluded that in the health-promoting network, numerous opportunities for further integration and interaction of health actors would exist at universities. Kellner et al. introduced the “house of studyability” which may be used as an orientation in the development of processes and sustainable structures. Last but not least, a systematic review including 21 studies by Kühn et al. provided an overview of studies examining health literacy among university students. The majority of studies reported health literacy scores among university students were lower compared to reference samples.

## Conclusion

The papers of this Research Topic cover a wide range of topics around university students' health including empirical, methodological, and conceptual papers, studies evaluating health promotion interventions as well as a systematic review. However, most of the included studies are from German or European research groups what may be due to the fact that potential contributing authors were contacted using the personal network of the handling editors of this Research Topic. Although the results of this Research Topic might have limited generalizability from a global perspective, the contributions address the lack of research in this research field in most European countries as concluded in a recent systematic umbrella review (5). In order to gain a balanced global view in public health promotion in university students, future contributions focusing on to previously underrepresented regions are desirable.

## Author contributions

All authors listed have made a substantial, direct, and intellectual contribution to the work and approved it for publication.

## Conflict of interest

The authors declare that the editorial was conducted in the absence of any commercial or financial relationships that could be construed as a potential conflict of interest.

## Publisher's note

All claims expressed in this article are solely those of the authors and do not necessarily represent those of their affiliated organizations, or those of the publisher, the editors and the reviewers. Any product that may be evaluated in this article, or claim that may be made by its manufacturer, is not guaranteed or endorsed by the publisher.
